# A Complicated Pregnancy in an Adult with HNF4A p.R63W-Associated Fanconi Syndrome

**DOI:** 10.1155/2019/2349470

**Published:** 2019-12-25

**Authors:** Oluwaseun Anyiam, Elizabeth Wallin, Felicity Kaplan, Christopher Lawrence

**Affiliations:** ^1^Imperial College London, London, UK; ^2^East and North Hertfordshire NHS Trust, Stevenage, UK

## Abstract

Renal Fanconi syndrome (RFS) is characterised by generalised dysfunction of the proximal renal tubules, resulting in excessive urinary loss of solutes, most notably bicarbonate, and type II (proximal) renal tubular acidosis. It is a rare condition, and literature around its management through pregnancy is limited. We present the management of a 37-year-old woman with RFS secondary to the HNF4A p.R63W mutation, through her third pregnancy. She presented at 28 + 5 weeks with dehydration, low serum bicarbonate, and profound metabolic acidosis. Daily infusions of sodium bicarbonate were necessary, and the requirements increased throughout the pregnancy. She also demonstrated both fasting hypoglycaemia and episodes of postprandial hyperglycaemia which required complex management. Due to concerns around fetal health, an elective caesarean section was performed at 34 weeks, delivering a healthy baby girl. This case highlights the potential complexity of pregnancy in patients with RFS and the need for a multidisciplinary approach to its management.

## 1. Introduction

Renal Fanconi syndrome (RFS) is a disorder characterised by proximal tubular dysfunction, leading to impaired reabsorption of filtered substances including phosphate, glucose, and bicarbonate [1, 2]. Clinical features include hypophosphataemia, hypokalaemia, and renal tubular acidosis [1], although the presentation of the condition is highly variable, particularly in states of physiological stress.

Pregnancy also physiologically modulates renal function, resulting in increased glomerular filtration rate (GFR) and compensatory rises in tubular reabsorption [3]. Glycosuria and mild proteinuria also occur. In addition, hyperventilation of pregnancy results in a mild respiratory alkalosis, and in the normal kidney, bicarbonate reabsorption is reduced to compensate for the respiratory changes [4].

Careful medical treatment of children with RFS and other chronic renal disorders is improving outcomes [5, 6] meaning that more are reaching childbearing age without significant disability and are thus conceiving successfully. An awareness of how this syndrome interacts with the physiological changes seen in pregnancy is therefore important to allow clinicians to manage these potentially complex patients effectively during pregnancy.

We report a pregnancy in a patient with RFS secondary to the hepatocyte nuclear factor 4*α* (HNF4A) p.R63W mutation and the management required through to delivery of a healthy baby girl.

### 1.1. Case Presentation

A 37-year-old woman presented at 28 + 5 weeks' gestation with persistent vomiting and reduced oral intake. She had a background of primary RFS, diagnosed at the age of 3 years old when she presented with rickets. She also had a long history of progressive renal insufficiency, persistent proteinuria, nephrocalcinosis, and recurrent nephrolithiasis. Additionally, she had a chronic right-sided hydronephrosis from a previous episode of nephrolithiasis and was under regular nephrology follow-up receiving oral solute replacement (see Table 1).

She had two previous pregnancies, both monitored regularly due to her chronic kidney disease (CKD) and both delivered preterm due to placental abruption. Whilst her first pregnancy was uncomplicated until the sudden delivery at 37 weeks, the second pregnancy had been complicated by gestational diabetes, recurrent hypoglycaemia, and metabolic acidosis, which required occasional intravenous bicarbonate replacement. Following delivery at 30 weeks, the baby developed neonatal hypoglycaemia which prompted genetic testing, resulting in discovery of the HNF4A p.R63W genetic mutation in both mother and baby. Sadly, the child died at 6 months of age, possibly due to metabolic complications of the disease.

During her third pregnancy, she was being seen regularly in both the consultant-led antenatal clinic and the nephrology clinic. At her booking appointment, she was normotensive with a body mass index of 27 kg/m^2^. Serum bicarbonate and creatinine were stable within her usual range at 21 mmol/l and 190 *µ*mol/l, respectively, and urine protein/creatinine ratio was elevated but also stable for her at 223 mg/mmol. During early pregnancy, her bicarbonate levels had dropped as nausea prevented her from maintaining adequate oral replacement. Because of this, the decision had been made at 24 weeks to commence fortnightly bicarbonate infusions via the day case unit to manage her persistent metabolic acidosis. As in her second pregnancy, she had developed gestational diabetes (Table 2) and therefore, was also under regular review in the joint diabetes antenatal clinic.

Despite the infusions, her nausea progressed to persistent vomiting and she was unable to tolerate any oral solute therapy. On admission at 28 + 5 weeks, she was dehydrated with cool peripheries and dry mucous membranes. She was hypotensive at 89/50 mmHg, acidotic with low bicarbonate, and had borderline phosphate, potassium, and glucose levels (see Table 3). Her urea and creatinine levels were at her baseline. There was concern that she may be developing HELLP syndrome as her alanine transferase (ALT) levels were elevated; however, her platelet count was greater than 100 × 10^9^/l and haemolysis screen was negative.

### 1.2. Treatment and Follow-Up

She was admitted for intravenous bicarbonate replacement which rapidly corrected her acidaemia but this relapsed upon cessation of the infusion. She also developed recurrent symptomatic hypoglycaemia and hypokalaemia, necessitating intravenous replacement.

To maintain normoglycaemia, normokalaemia, and normal serum pH, she required a daily replacement regimen comprising 500–1000 ml 1.26% sodium bicarbonate, 1000 ml 5% dextrose with additional potassium replacement as required, and a high carbohydrate diet. The daily fluid regimen combined with the need for daily blood tests meant that she remained an inpatient for the remainder of her pregnancy. Her ALT levels improved through the course of the admission, and an abdominal ultrasound demonstrated patent hepatic and portal veins with no identifiable liver lesion.

In discussion with the patient, to prevent metabolic complications for either the mother or the baby, the obstetricians planned an elective caesarean section at 34 weeks' gestation with fortnightly fetal scans until delivery. At 30 weeks, two doses of intramuscular dexamethasone were administered to aid fetal lung maturation. In the two days following these doses, the patient had no episodes of hypoglycaemia and in fact developed episodes of postprandial hyperglycaemia, so dextrose infusions were suspended. However, once the effects of the steroids had worn off, her symptomatic hypoglycaemia returned, and dextrose infusions were required once again.

From 31 weeks' gestation, her serum potassium began to rise, however bicarbonate levels continued to fall. Thus, bicarbonate infusions were correspondingly increased, and intravenous potassium replacement was ceased. By 34 weeks, she was requiring 2 litres per day of 1.26% sodium bicarbonate. Throughout the inpatient stay, her serum creatinine remained stable.

At 34 weeks, a baby girl was delivered by elective caesarean section. The birth weight was reasonable for gestation at 2,310 g, and Apgar scores were 9 and 10 at five and ten minutes, respectively. She was transferred to the special care babies' unit for further observation. Following delivery, the mother's biochemical abnormalities resolved within three days. She was monitored for 5 days postcaesarean, but as all electrolytes were stable off intravenous replacement, she was discharged on her previous doses of oral solute replacement.

She continues under follow-up in the nephrology clinic with stable renal function that has not declined from prepregnancy levels, despite the complications of her third pregnancy. Moreover, her gestational diabetes resolved and her glycosylated haemoglobin (HbA1c) level remains within the normal range.

## 2. Discussion

Renal Fanconi syndrome (RFS) is characterised by generalised dysfunction of the proximal renal tubules resulting in impaired reabsorption and therefore increased urinary excretion of solutes [1, 2]. This can lead to a variety of clinical manifestations including dehydration, polyuria, hypokalaemia, hypophosphataemic rickets (or osteomalacia in adults), and impaired growth [1]. Renal insufficiency and metabolic acidosis secondary to type II (proximal) renal tubular acidosis, resulting from excessive urinary losses of bicarbonate, can also occur. The clinical presentation of RFS shows considerable variability but it should be considered in a patient presenting with hypokalaemia, hypophosphataemia, low serum bicarbonate, and metabolic acidosis. It is confirmed by demonstrating increased urinary loss of potassium, phosphate, bicarbonate, glucose, amino acids, and low molecular weight proteins. Treatment is usually supportive with oral replacement of potassium, bicarbonate, phosphate, and activated vitamin D [7].

RFS can be inherited or acquired. Acquired causes include multiple myeloma, amyloidosis, and drugs, particularly antiretrovirals, antiepileptics, aminoglycoside antibiotics, and platinum-containing chemotherapy agents [8, 9]. Inherited RFS is generally associated with systemic conditions arising from specific gene mutations with cystinosis being by far the most common (see Table 4) [8, 10].

Recently, a mutation in the HNF4A gene has been identified as a rare cause of inherited RFS. The p.R63W mutation of the HNF4A gene was first described by Hamilton et al. in 2014 and results in a combination of RFS and the pancreatic *β*-cell dysfunction seen in mature onset diabetes of the young (MODY) [11]. The reported features include macrosomia and neonatal hypoglycaemia in infancy, with the onset of RFS in childhood and an increased risk of diabetes mellitus in adulthood [11]. Nephrocalcinosis and liver dysfunction are additional manifestations of this mutation [12, 13]. There are few case reports in the literature [12–15], and to our knowledge, there are no reports of patients with this mutation presenting during pregnancy.

We describe the case of a 37-year-old woman with RFS secondary to the HNF4A p.R63W mutation. Her history and the complications of her previous pregnancies made her case particularly challenging. Firstly, maintenance of stable plasma levels of solutes and normal serum pH was essential as severe prolonged metabolic acidosis in pregnancy is known to impair fetal circulation and cause fetal distress [16]. Early in gestation, outpatient intravenous bicarbonate infusions were commenced, in addition to oral supplementation, to achieve this. However, her inability to maintain oral supplementation due to a mild vomiting illness led to rapid development of a profound metabolic acidosis, necessitating admission for intensive intravenous bicarbonate replacement.

Her bicarbonate requirements appeared to increase throughout gestation, and thus, by the time of delivery, the daily bicarbonate requirement had increased four-fold. It is well known that additional supplementation of bicarbonate can exacerbate urinary losses of both bicarbonate and potassium in RFS. However, this had to be balanced against the need to maintain adequate serum bicarbonate levels and avoid symptomatic acidaemia. Therefore, rather than aiming to normalise serum bicarbonate levels (resulting in further losses), the aim was to maintain levels of 17–20 mmol/l. Interestingly, potassium levels stabilised by 31 weeks, despite infusing increasing amounts of bicarbonate. The phenomenon of rising bicarbonate requirements through gestation has not been previously described in patients with RFS, although it has been noted in other causes of renal tubular acidosis [17, 18].

Another challenging aspect of this case was maintaining euglycaemia. This patient clearly demonstrated evidence of *β*-cell dysfunction with an abnormal glucose tolerance test, leading to the diagnosis of gestational diabetes. However, she also suffered recurrent episodes of symptomatic hypoglycaemia requiring management with a high carbohydrate diet and intravenous glucose replacement. Furthermore, when steroids were administered at 30 week's gestation, the consequent increase in insulin resistance, in conjunction with the underlying pancreatic dysfunction, resulted in hyperglycaemia.

The recurrent hypoglycaemia could not be easily explained but one postulated theory was increased urinary glucose loss due to the combination of pregnancy-associated changes and RFS. This is supported by the fact that her symptoms resolved immediately postpartum. However, it is important to note that no urinary glucose excretion level was available to confirm this, and whilst glycosuria is a well-recognised feature of RFS, it does not usually result in hypoglycaemic episodes [8, 9].

Concurrent pancreatic *β*-cell dysfunction is only described in cases of the HNF4A p.R63W mutation and in Fanconi–Bickel syndrome, another rare genetic cause of RFS [19]. The paradoxical coexistence of fasting hypoglycaemia and postprandial hyperglycaemia in RFS seems to be a unique feature of these two syndromes and appears to become more clinically manifest during pregnancy. This made achieving euglycaemia a difficult challenge, again highlighting how precarious the management of electrolyte balance was in this case.

The plan for delivery was agreed following discussion between the patient and the involved clinicians as several issues needed careful consideration. Whilst it is well recognised that preterm delivery is associated with increased risk of morbidity [20], there is also an increased risk of spontaneous preterm delivery in patients with CKD [21]. Accordingly, preexisting CKD is an indication for planned preterm delivery [22]. Our patient already had a history of preterm placental abruption, which significantly increases the risk of subsequent deliveries occurring at an earlier gestational age [23]. The potential effects of prolonged acidosis on the developing fetus also had to be recognised. Finally, the patient had significant concerns about delaying delivery, given the outcome of her previous pregnancies. When all factors were considered, the collaborative decision was made to arrange for elective caesarean section at 34 weeks' gestation.

It is not clear why the clinical course seemed to worsen with each subsequent pregnancy in this patient. This has previously been reported in patients with CKD [18, 24, 25], suggesting further investigation into this phenomenon is required. However, it is important to acknowledge that in this group of patients, a previously uncomplicated pregnancy does not eliminate the risk of subsequent pregnancies developing complications. This emphasises the need for pregnancies in women with preexisting CKD to be managed as high risk, in line with current recommendations [21, 26]. This involves regular antenatal follow-up by both obstetricians and renal physicians and monitoring of all markers of renal function, including blood pressure, fluid status, urine protein excretion, and serum electrolytes.

In summary, we report the case of a 37-year-old woman with RFS caused by the rare HNF4A p.R63W mutation and the management of electrolyte imbalances through her pregnancy, which have not previously been described. Maintenance of safe plasma solute levels and euglycaemia was highly challenging but crucial to preventing perinatal morbidity. Through careful monitoring and early planning of delivery, it was possible to support her through pregnancy and safe delivery of a healthy baby at 34 weeks' gestation.

As medicine continues to advance and more women with CKD consider pregnancy, the number of pregnant women with rare conditions such as RFS will increase. A wider understanding of these conditions will both encourage more informed decision-making and prevent additional concern for patients during this already stressful time. We hope that this report will help raise awareness of the HNF4A p.R63W mutation, as well as other causes of RFS, and promote further discussion about management of patients with RFS safely through pregnancy to successful delivery.

## Figures and Tables

**Table 1:** Preadmission medication list.

(i) Alfacalcidol 0.75 *μ*g once daily
(ii) Sodium bicarbonate 8 g twice daily
(iii) Phosphate Sandoz 2 tablets morning, 1 tablet evening
(iv) Potassium citrate 30 ml twice daily
(v) Ferrous sulphate 200 mg twice daily

**Table 2:** Glucose tolerance test results.

Fasting	3.8 mmol/l
Post-oral glucose load	11.0 mmol/l

**Table 3:** Admission blood results.

	Result	Normal ranges
Sodium	134 mmol/l	133–146
Potassium	3.5 mmol/l	3.5–5.3
Urea	4.0 mmol/l	2.5–7.8
Creatinine	186 *μ*mol/l	45–84
Bicarbonate	16 mmol/l	22–29
Chloride	106 mmol/l	95–108
Adjusted calcium	2.27 mmol/l	2.2–2.6
Magnesium	0.97 mmol/l	0.7–1.0
Inorganic phosphate	0.84 mmol/l	0.8–1.5
Random glucose	3.9 mmol/l	3.5–8
pH	7.28	7.35–7.45
Base excess	−9.1	−2.0–2.0
Lactate	1.23 mmol/l	0–2.0
Bilirubin	8 *μ*mol/l	0–21
Alkaline phosphatase	78 IU/l	30–130
Alanine transferase	129 IU/l	7–40
Albumin	39 g/L	35–50
Platelets	127 × 10^9^/l	150–400
Lactate dehydrogenase	139 IU/l	135–225

**Table 4:** Some causes of inherited RFS [8, 10].

(i) Cystinosis
(ii) Galactosaemia
(iii) Tyrosinaemia
(iv) Wilson's disease
(v) Lowe's syndrome
(vi) Hereditary fructose intolerance

## Additional Points

(i) Renal Fanconi syndrome (RFS) results from generalised dysfunction of renal proximal tubules causing excessive urinary loss of essential solutes and type II renal tubular acidosis. (ii) The HNF4A p.R63W gene mutation results in a combination of RFS and pancreatic *β*-cell dysfunction with additional features such as liver dysfunction. (iii) RFS can worsen during pregnancy and patients with severe cases may require admission for careful intravenous solute replacement. (iv) Management of patients with CKD during pregnancy is challenging, it carries greater maternal and perinatal risks, and regular monitoring by a specialist multidisciplinary team is essential.
